# Techniques to Teach Students Effectively Using Telemedicine. Comment on “Incorporating Medical Students Into Primary Care Telehealth Visits: Tutorial”

**DOI:** 10.2196/30703

**Published:** 2022-03-11

**Authors:** Hardeep Kandola, Sonica Minhas

**Affiliations:** 1 Barts and the London School of Medicine and Dentistry Queen Mary University of London London United Kingdom

**Keywords:** medical student, education, primary care, telehealth, video visits, internal medicine, medical education, teleconsultation, digital health, COVID-19, teaching, telemedicine, clerkships

We read the findings of Balaji and Clever [1] with great interest, which highlight a successful approach to engaging students with patients on community-based placements during a challenging public health crisis. While we acknowledge the limited sample size of this study, our experience as senior clinical medical students also reflects the merits of telemedicine for continuing medical education [2] where direct patient care has been limited.

We endorse the suggested recommendations as highly effective in practice based on our experience. We wish to propose further recommendations from our personal observations. First, prior to a consultation, providing students access to the patient’s presenting complaint and their medical history leads to a focused consultation. Patients often redirect clinicians to check their records when asked questions about their background. Accessing patient details beforehand can save valuable time and facilitate rapport building. Furthermore, where students have just started their placement or clerkship, or clinicians are unfamiliar with their assignee, a “see one, do one, teach one” or “knows how, shows how, does” experiential learning approach is recommended [3,4]. In lieu of teaching, students will hopefully be able to conduct history-taking autonomously, with minimal supervision. First, observing an interaction can build familiarity and orientation with software, and can help set the clinicians’ expectations and reduce student anxiety. Next, observing the student interaction allows the clinician to allay any safety concerns while suggesting improvements in manner and approach.

We suggest an alternative approach to enhance the value students can provide to the general practitioner (GP). We propose having students call patients during a fixed time in the morning and discussing their presentations after appointments are scheduled; this includes a differential diagnosis and proposed management with the GP as well. In our experience, many patients are available and amenable to being called earlier than their appointment time to speak with a student once assured they will be speaking to a doctor later. GPs can then call the patient to confirm the history, ask additional questions, and finalize a management plan, including assessing the need for an in-person follow-up.

Telemedicine has been rapidly adopted as a means of providing remote care, protecting patients and health care providers from direct infection transmission. Since its integration into daily practice, the convenience and cost savings for both patients and practitioners indicate that it is unlikely to disappear [5]. It is one successful avenue to continue students’ education and provide opportunities for engagement in patient care, in light of the disruption to clinical placements and face-to-face teaching. Digital competence and familiarity have become a vital part of the medical curriculum, meaning that students need to be trained to provide high-quality care through such technologies.

## Abbreviations

GP: general practitioner

## References

[1] Balaji A, Clever SL (2021). Incorporating Medical Students Into Primary Care Telehealth Visits: Tutorial. JMIR Med Educ.

[2] Abraham Heather N, Opara Ijeoma N, Dwaihy Renee L, Acuff Candace, Brauer Brittany, Nabaty Renieh, Levine Diane L (2020). Engaging Third-Year Medical Students on Their Internal Medicine Clerkship in Telehealth During COVID-19. Cureus.

[3] Kotsis Sandra V, Chung Kevin C (2013). Application of the "see one, do one, teach one" concept in surgical training. Plast Reconstr Surg.

[4] Ten Cate Olle, Carraccio Carol, Damodaran Arvin, Gofton Wade, Hamstra Stanley J, Hart Danielle E, Richardson Denyse, Ross Shelley, Schultz Karen, Warm Eric J, Whelan Alison J, Schumacher Daniel J (2021). Entrustment Decision Making: Extending Miller's Pyramid. Acad Med.

[5] Portnoy Jay, Waller Morgan, Elliott Tania (2020). Telemedicine in the Era of COVID-19. J Allergy Clin Immunol Pract.

